# Supplementary material for: Delocalized electronic engineering of TiNb2O7 enables low temperature capability for high-areal-capacity lithium-ion batteries
Source: Nat Commun. 2024 Jul 26;15:6299. doi: 10.1038/s41467-024-50455-1 (PMC11282191; doi:10.1038/s41467-024-50455-1)
Supplement: Supplementary file 1 — Supplementary Information [file 41467_2024_50455_MOESM1_ESM.pdf]

Supporting Information

Delocalized electronic engineering of $TiNb_2O_7$ enables low temperature capability for high-areal-capacity lithium-ion batteries

Yan Zhang[1#],*Yingjie Wang*[2#], *Wei Zhao*[1], *Pengjian Zuo*[1], *Yujin Tong*[3], *Geping Yin*[1]*, *Tong Zhu*[2]*, *Shuaifeng Lou*[1]*

1. State Key Laboratory of Space Power-Sources, Harbin Institute of Technology, Harbin 150001, China.
2. Laser Micro/Nano Fabrication Laboratory, School of Mechanical Engineering, Beijing Institute of Technology, Beijing 100081, China.
3. Faculty of Physics, Duisburg-Essen University, D-47057 Duisburg, Germany.

* Corresponding author.

E-mail: yingeping@hit.edu.cn (G. Yin); E-mail: Tongzhubit@bit.edu.cn (T. Zhu); E-mail: shuaifeng.lou@hit.edu.cn (S. Lou).

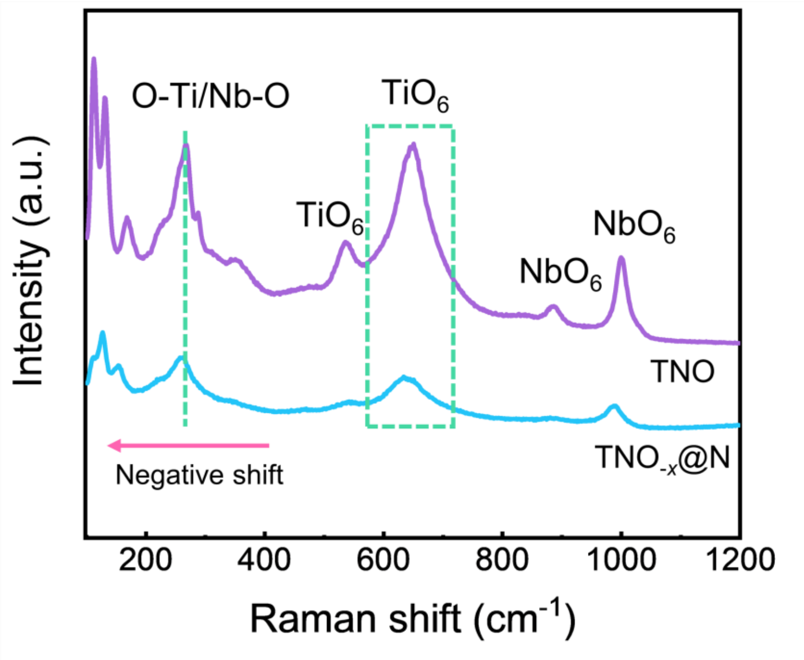

Supplementary Fig. 1. Raman spectra of the TNO and TNO$_{-x}$@N composite.

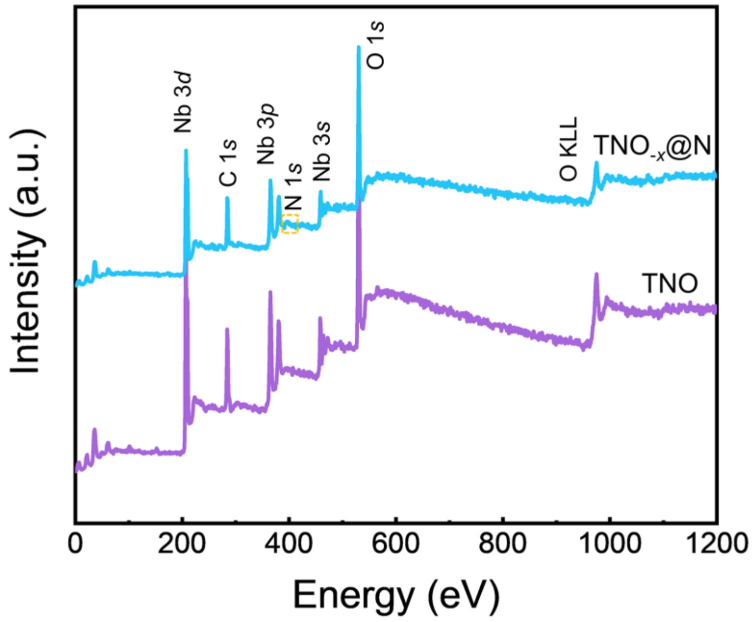

Supplementary Fig. 2. XPS survey spectrum of TNO and TNO$_{-x}$@N.

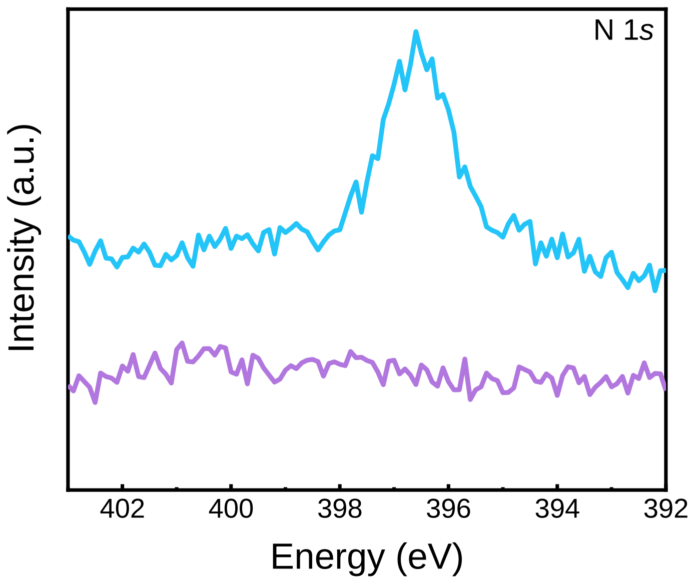

Supplementary Fig. 3. The deconvoluted N 1*s* of TNO and TNO$_{-x}$@N.

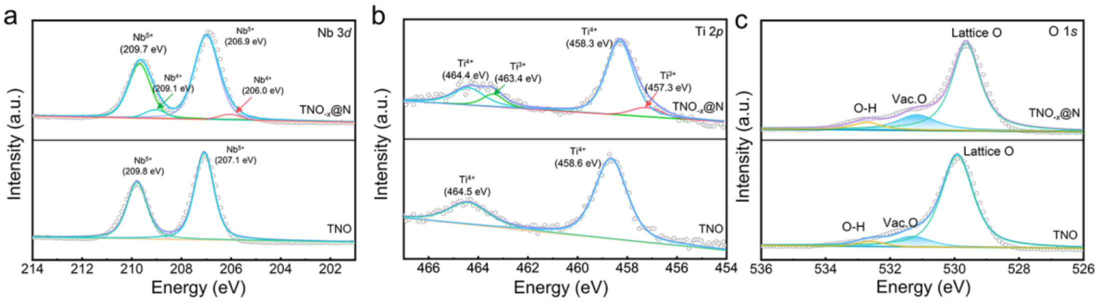

Supplementary Fig. 4. The deconvoluted Nb 3*d*, Ti 2*p*, and O 1*s* XPS spectra. XPS analysis spectra of (a) Nb 3*d*, (b) Ti 2*p*, and (c) O 1*s* of TNO and TNO_{-x}@N.

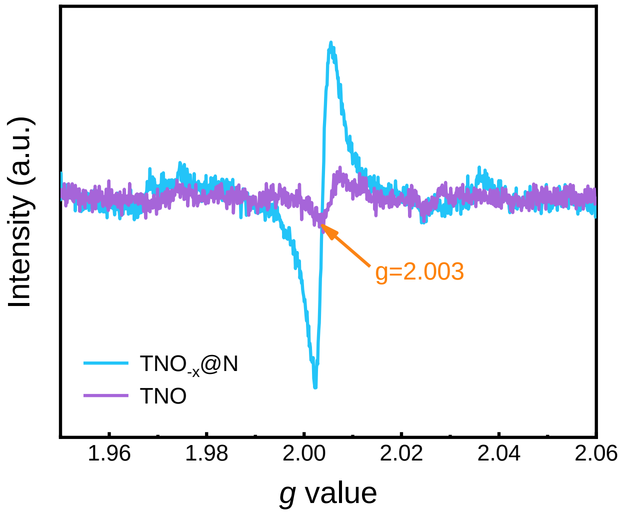

Supplementary Fig. 5. EPR spectra of TNO and TNO$_{-x}$@N.

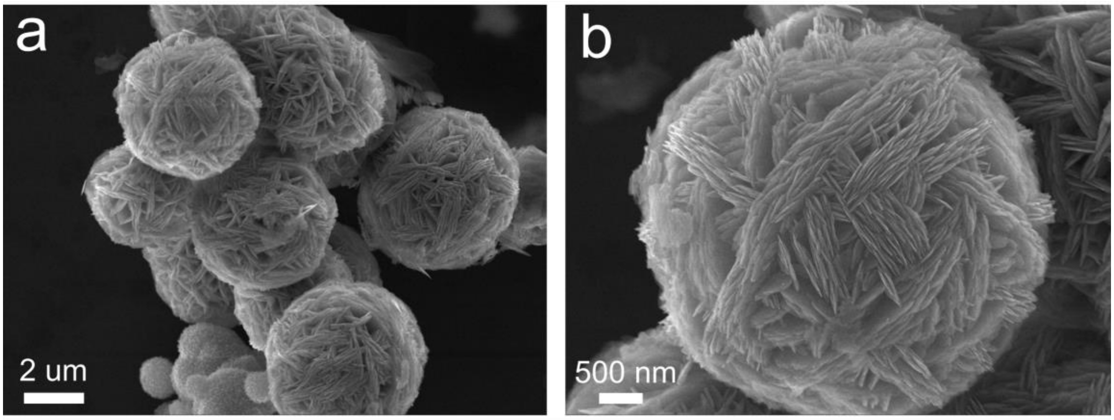

Supplementary Fig. 6. Morphology characterization of the TNO precursor. SEM images of the TNO precursor.

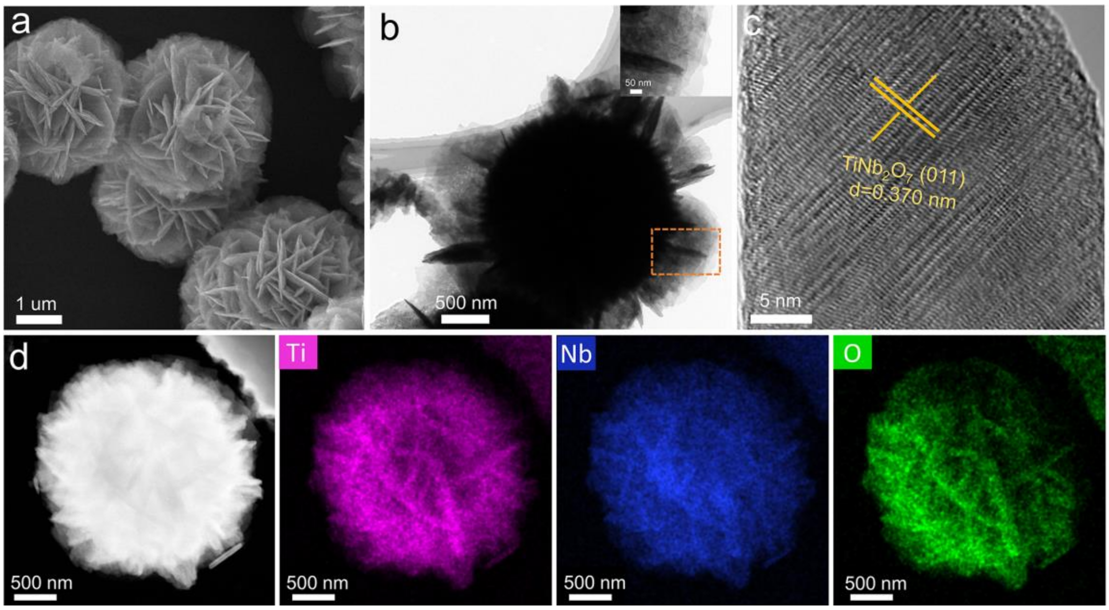

Supplementary Fig. 7. Morphology characterization of TNO. (a) SEM image, (b) TEM image, and (c) HRTEM image of TNO. (d) HAADF image and corresponding elemental mapping of TNO.

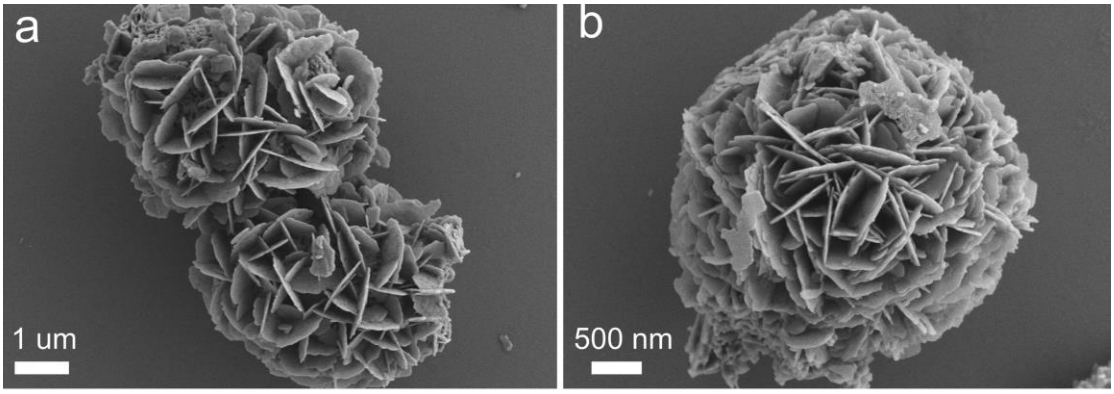

Supplementary Fig. 8. Morphology characterization of TNO$_{-x}$@N. SEM images of TNO$_{-x}$@N.

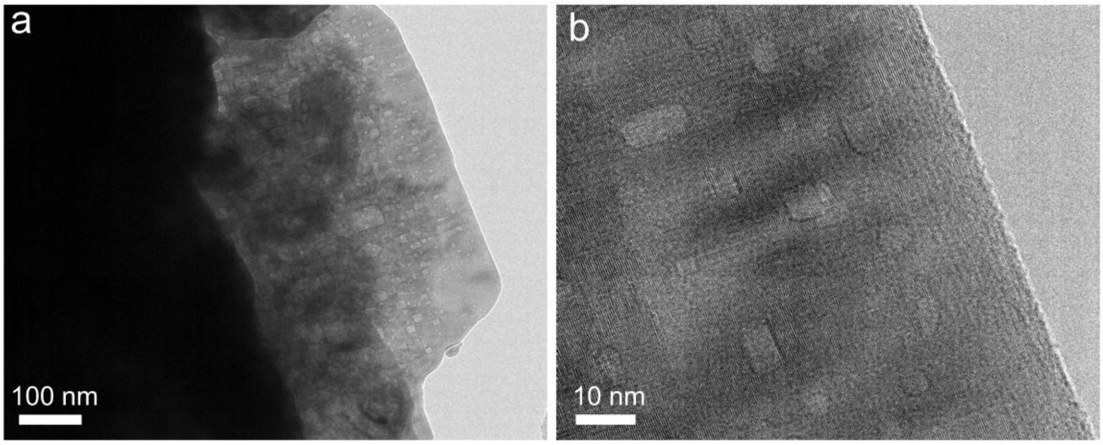

Supplementary Fig. 9. Characterization of compact TNO$_{-x}$@N microflowers. TEM images of TNO$_{-x}$@N.

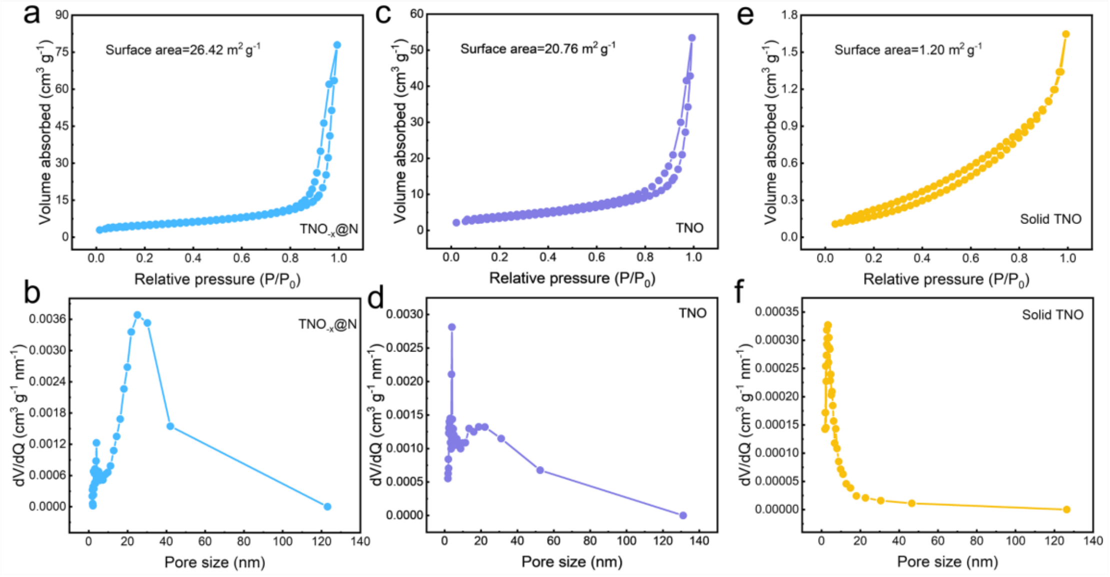

Supplementary Fig. 10. N_2 adsorption/desorption isotherms and pore size distribution. (a, b) TNO$_{-x}$@N, (c, d) TNO, and (e, f) solid TNO.

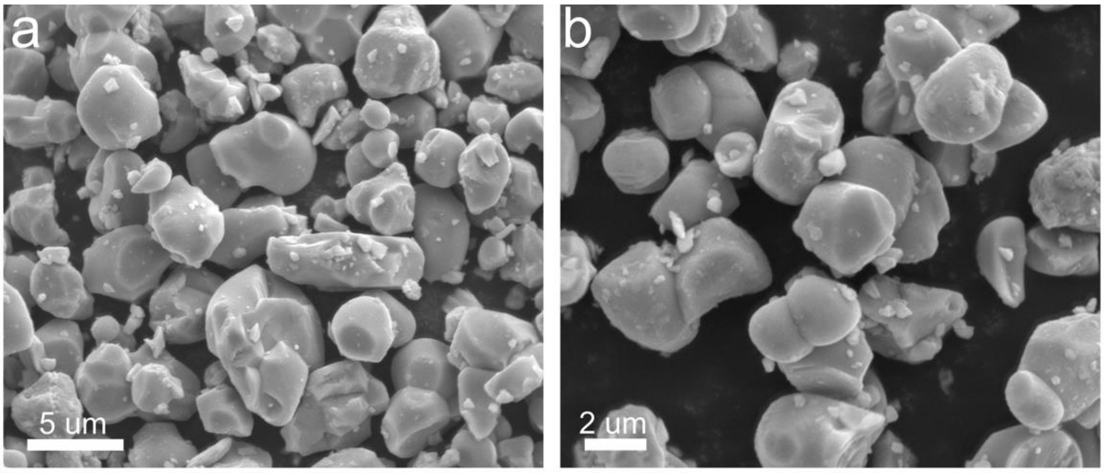

Supplementary Fig. 11. SEM images of the bulk TNO composite.

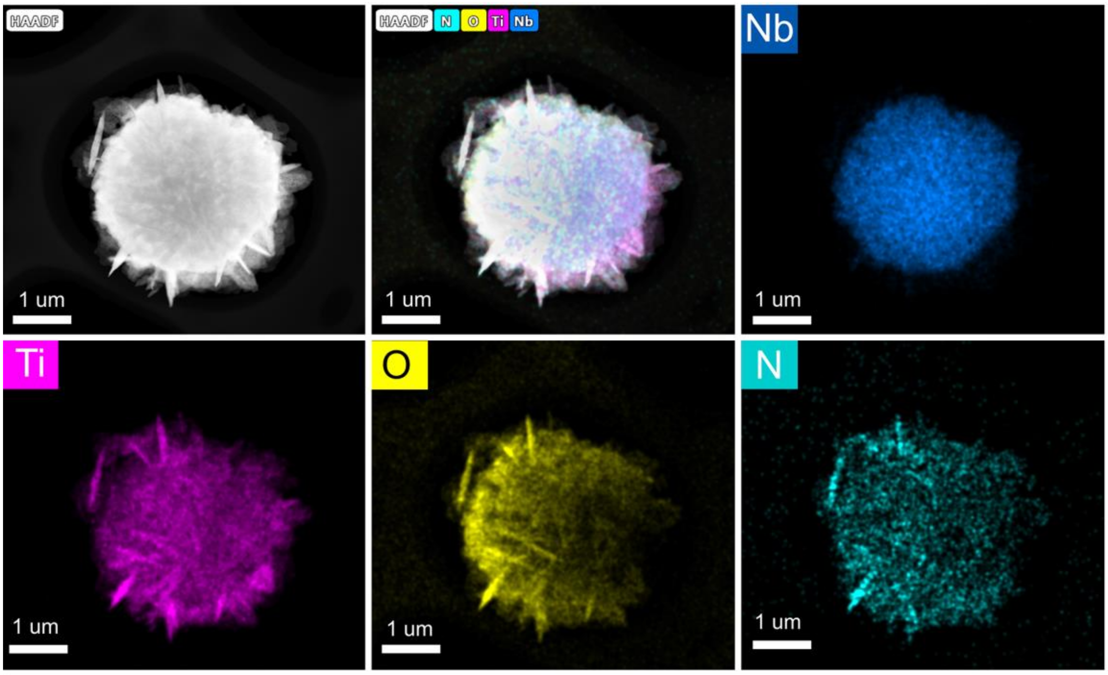

Supplementary Fig. 12. HAADF and corresponding elemental mapping images of TNO$_{-x}$@N.

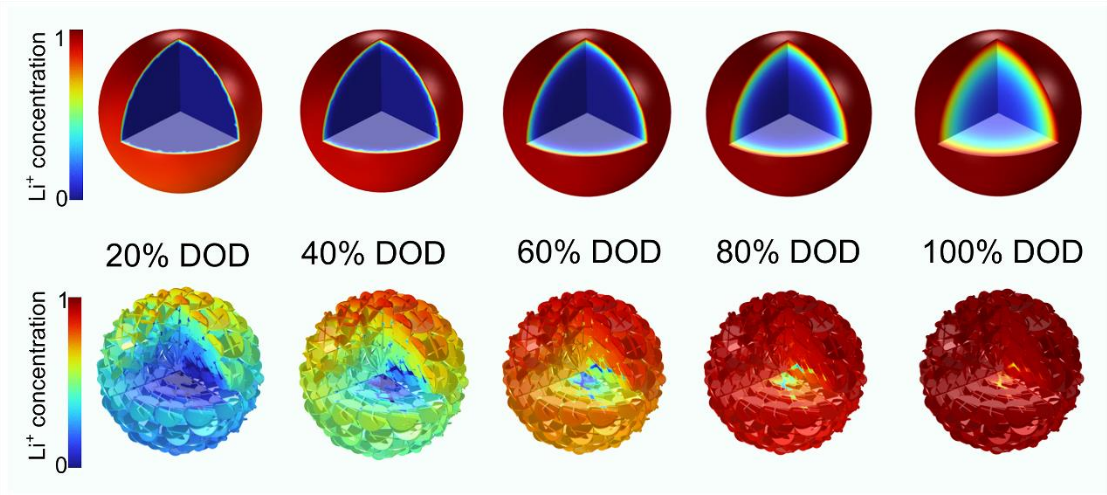

Supplementary Fig. 13. Finite element simulation models of Li^+ concentration of solid sphere and microflowers with the different lithiation states at the current density of 15 C.

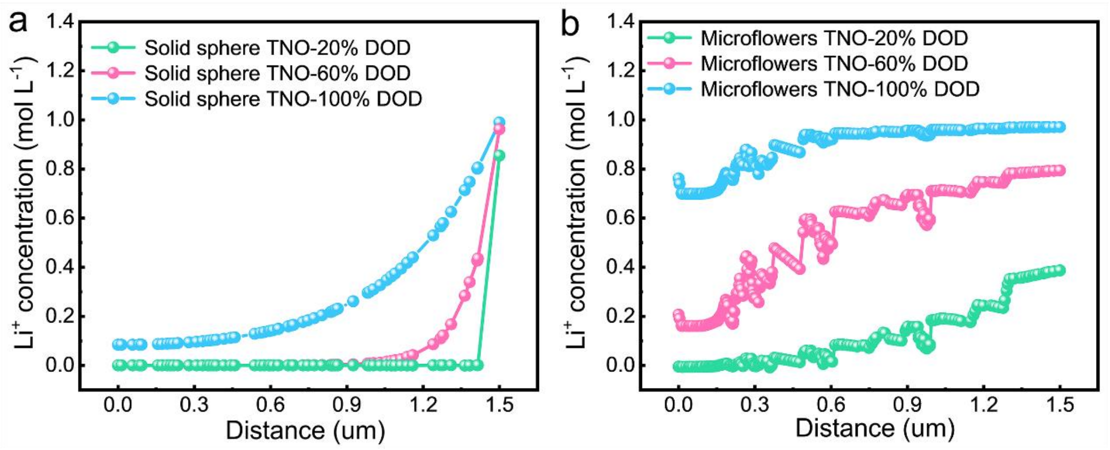

Supplementary Fig. 14. Evolution of Li^+ concentration in the (a) solid sphere and (b) microflowers at the current density of 15 C.

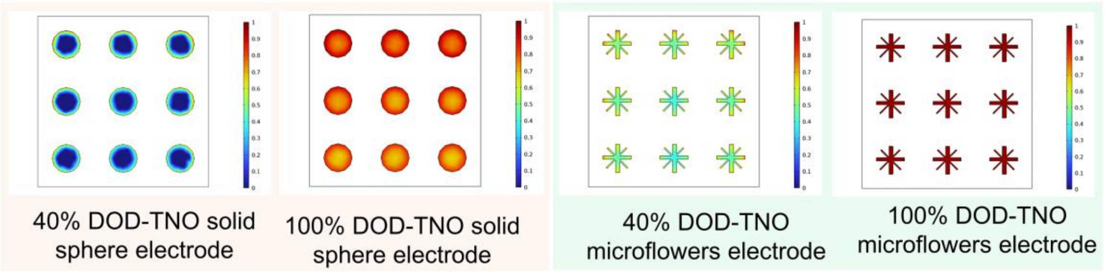

Supplementary Fig. 15. Li^+ concentration of solid sphere and microflowers at electrode level under the 40% DOD and 100% DOD state at 15 C.

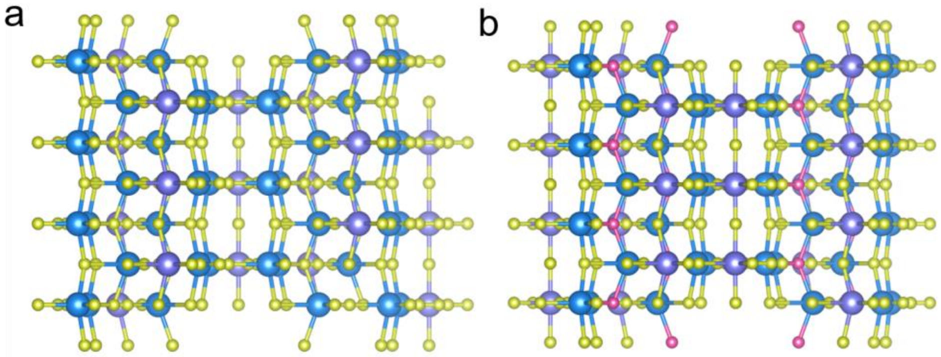

Supplementary Fig. 16. The optimized conformations of (a) TNO and (b) TNO@N. The yellow, purple, blue, and pink balls represent O, Ti, Nb, and N atoms, respectively.

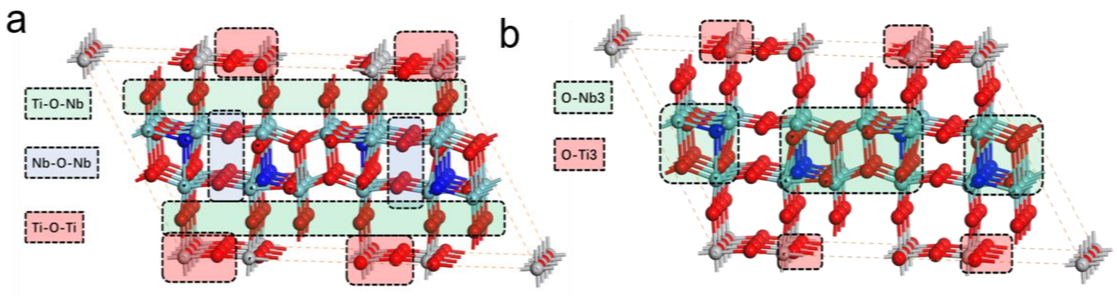

Supplementary Fig. 17. Screening of oxygen vacancy formation sites in TNO structures. Oxygen vacancy formation sites in TNO structures (a) dicoordination and (b) tricoordination.

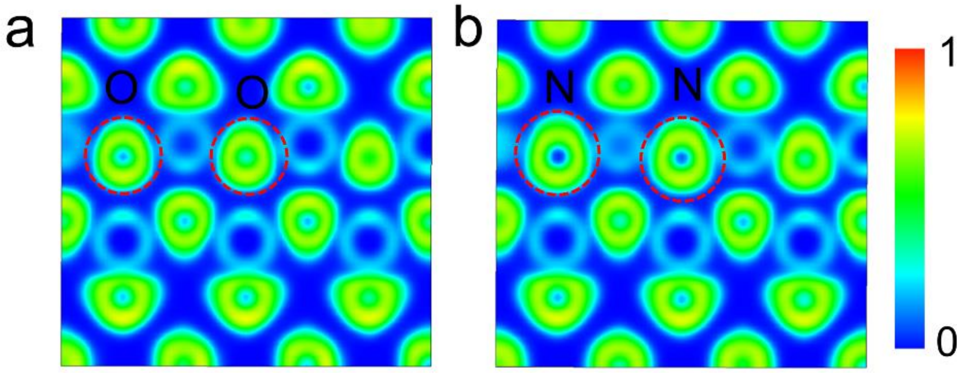

Supplementary Fig. 18. The ELF plots of (a) TNO and (b) TNO$_{-x}$@N.

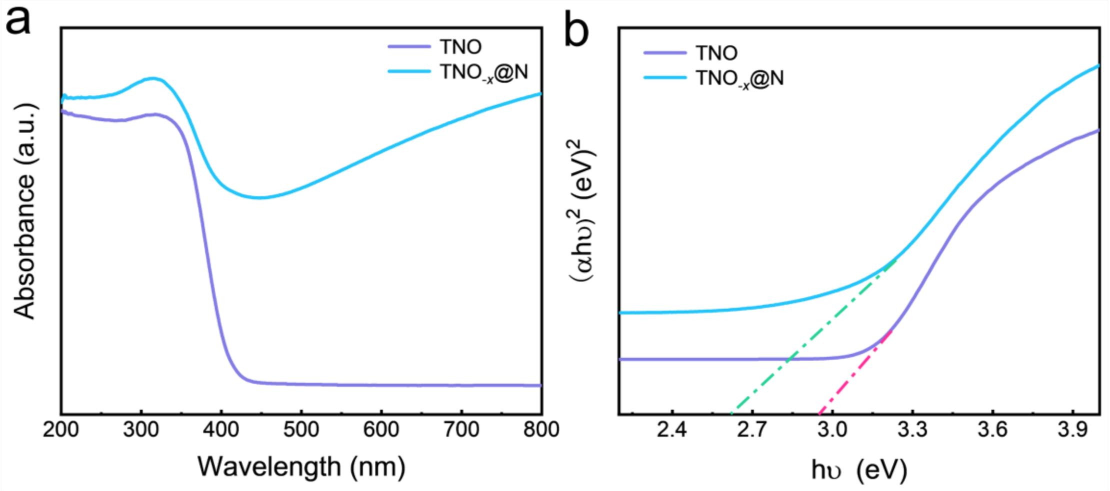

Supplementary Fig. 19. Ultraviolet–visible diffuse reflectance spectra (UV–vis DRS) of TNO and TNO$_{-x}$@N. (a) UV–vis. Absorption spectra of TNO and TNO$_{-x}$@N, (b) Relationship between (*αhv*) and photon energy demonstrating the band gap of TNO and TNO$_{-x}$@N.

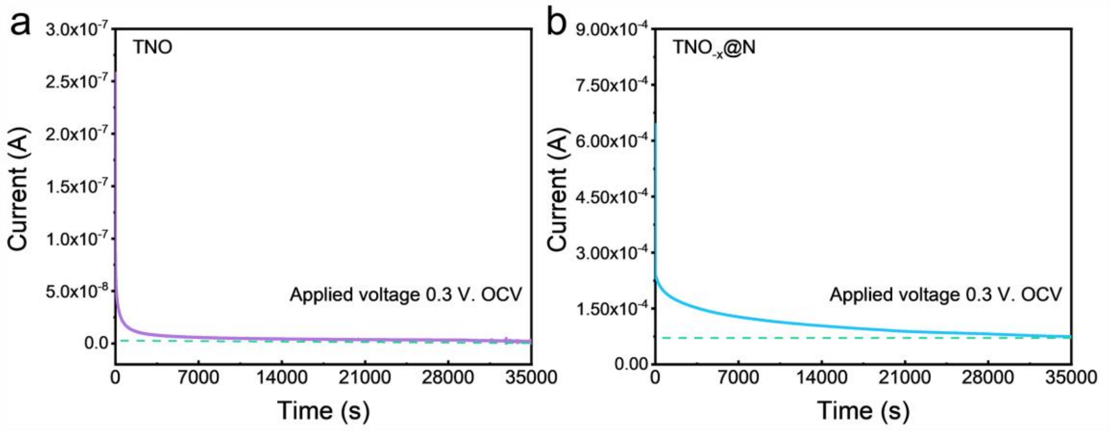

Supplementary Fig. 20. Direct polarization curve under an applied potential of 0.3 V at 25 °C. (a) TNO and (b) TNO$_{-x}$@N.

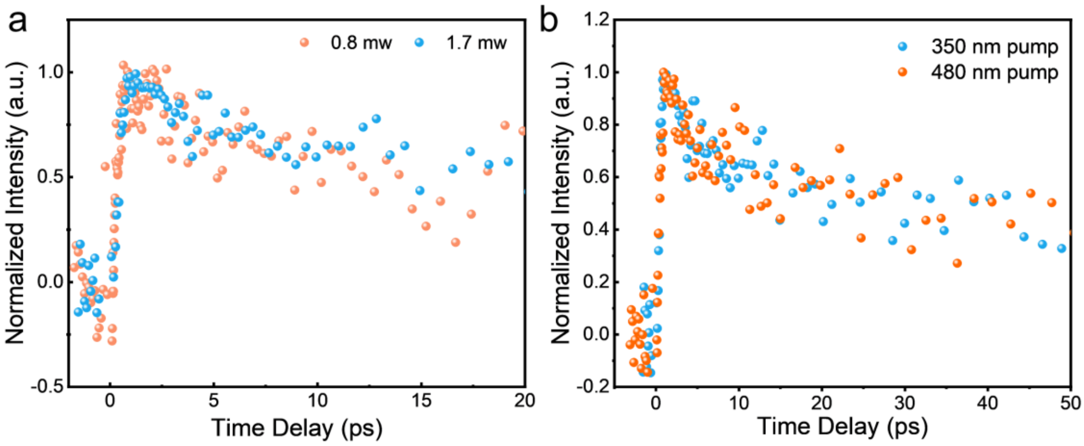

Supplementary Fig. 21. Dynamics comparison of TNO$_{-x}$@N at different excitation power and excitation energy. (a) TNO$_{-x}$@N TA spectra of 625 nm probe at excitation power of 0.8 mw cm^{-2} and 1.7 mw cm^{-2}. (b) Excitation energy dependent TA spectra of TNO$_{-x}$@N.

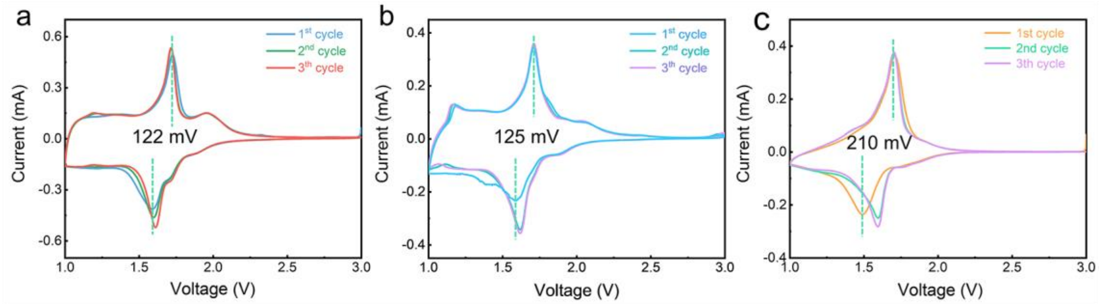

Supplementary Fig. 22. Electrochemical measurements of the TNO$_{-x}$@N, TNO and solid TNO. CV curves of (a) the TNO$_{-x}$@N, (b) TNO, and (c) solid TNO electrode for the first three cycles at a scan rate of 0.1 mV s^{-1} at 25 ^{o}C.

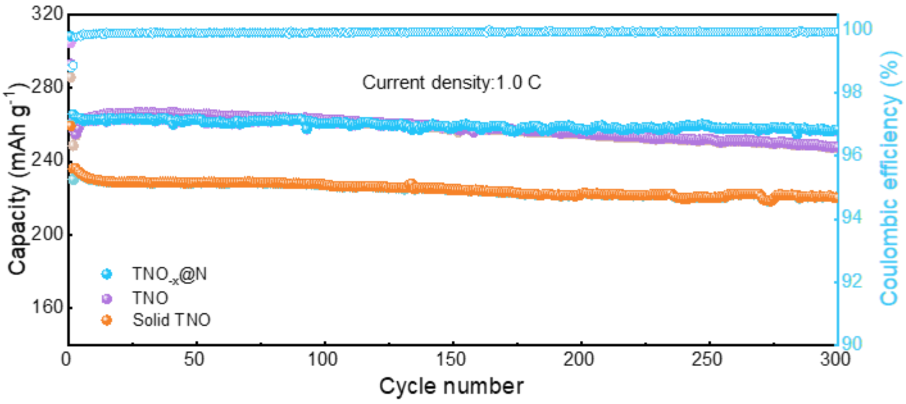

Supplementary Fig. 23. Cyclic performance of the TNO$_{-x}$@N, TNO and solid TNO operated at 1 C and 25 ^{o}C.

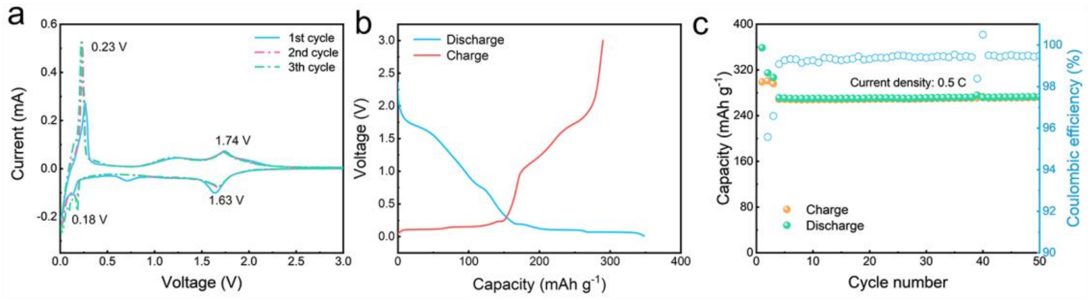

Supplementary Fig. 24. Electrochemical properties of the G/TNO electrode at 25 °C. (a) CV profiles of the G/TNO electrode at 0.1 mVs^{-1} within the potential range of 0.01–3 V during the initial 3 cycles. (b) Galvanostatic charge/discharge of the G/TNO electrode at 0.2 C. (c) Cyclic performance of the G/TNO electrode at 0.5 C. (1 C=350 mA g^{-1})

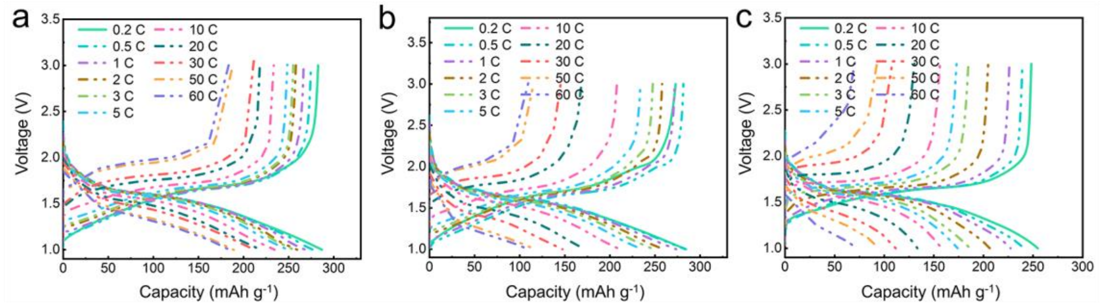

Supplementary Fig. 25. The rate capability of the TNO$_{-x}$@N, TNO and solid TNO. Discharge/charge curves of the TNO$_{-x}$@N, TNO and solid TNO composite at various current densities from 0.2 C to 60 C.

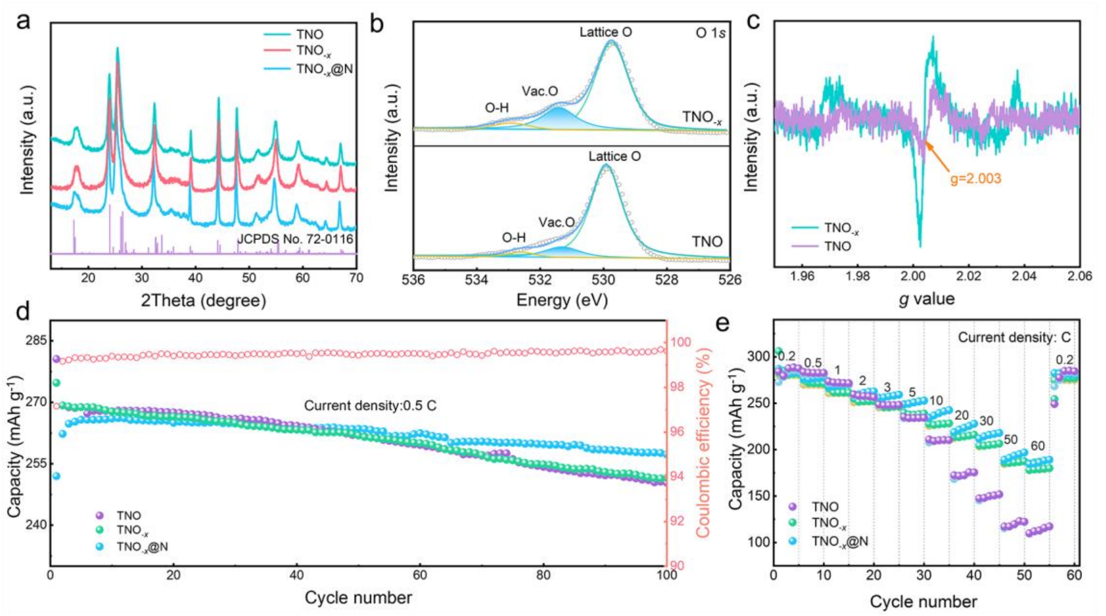

Supplementary Fig. 26. Structural characterization and electrochemical properties of TNO_{-x}. (a) XRD patterns of TNO, TNO_{-x} and TNO_{-x}@N; (b) High-resolution XPS O 1*s* spectra of TNO and TNO_{-x}; (c) EPR spectra of TNO and TNO_{-x}; (d) Cycling performance of the TNO, TNO_{-x} and TNO_{-x}@N electrode at 0.5 C; (e) Rate capability the TNO, TNO_{-x} and TNO_{-x}@N electrode. The mass loading of the electrode is about 1.5 mg cm^{-2}. (1 C=300 mA g^{-1})

From XRD in **Supplementary Fig. 26a**, the characteristic peaks of the TNO_{-x} sample are similar to the TNO and TNO_{-x}@N sample, which can be well indexed as the monoclinic phase of $TiNb_2O_7$ (JCDPS No. 72-0116), confirming the formation of O-vacancies does not change the bulk structure of $TiNb_2O_7$. To investigate the successful synthesis of oxygen defects in TNO_{-x}, X-ray photoelectron spectroscopy are investigated. As displayed in **Supplementary Fig. 26b**, the O 1*s* peaks in TNO_{-x} are fitted with three peaks at 529.7 eV, 531.4 eV, and 532.9 eV, which are attributed to the lattice O (metal−oxygen bands), oxygen vacancies, and oxygen species in hydroxyl oxygen, respectively. The peak area ratio of O vacancy to lattice O for TNO_{-x} (0.28) is much higher than that of TNO (0.12), which indicates that the reducing atmosphere can effectively trigger the lattice oxygen loss. Further analysis based on electron paramagnetic resonance (EPR) confirm the XPS results. In **Supplementary Fig. 26c**, TNO_{-x} displays an obvious oxygen vacancy signal peak at g=2.003, whereas TNO exhibits only a slight resonance at this position, which further confirms the presence of bulk-phase oxygen vacancies in TNO_{-x} in addition to surface vacancies.

The cycling performances of the TNO, TNO_{-x} and TNO_{-x}@N electrode are compared and the results are shown in **Supplementary Fig. 26d**. The TNO_{-x} electrode delivers the specific capacity of 251.6 mAh g^{-1} after 100 cycles at 0.5 C, which is preferred to that of pure TNO flower (250.4 mAh g^{-1}), but lower than that of TNO_{-x}@N (257.8 mAh g^{-1}). This result suggests that the O-vacancies feature does not significantly improve the cycling stability of the TNO material, and therefore the N doping feature is the main reason for the excellent cycling performance. **Supplementary Fig. 26e** further compares the fast charging capability of the TNO, TNO_{-x} and TNO_{-x}@N electrode in the current density range from 0.2 to 60 C. The TNO_{-x} electrode delivers a capacity of 179.1 mAh g^{-1} at a high current density 60 C, which far exceeds that of TNO (113.6 mAh g^{-1}), approaching TNO_{-x}@N (186.7 mAh g^{-1}). This result indicates that the O-vacancies feature is the main contribution to the improvement of the rate performances of the TNO material, which may be due to O-vacancies can provide abundant active sites to accelerate near-surface reaction, leading to the more obvious intercalation pseudo-capacitance behavior.

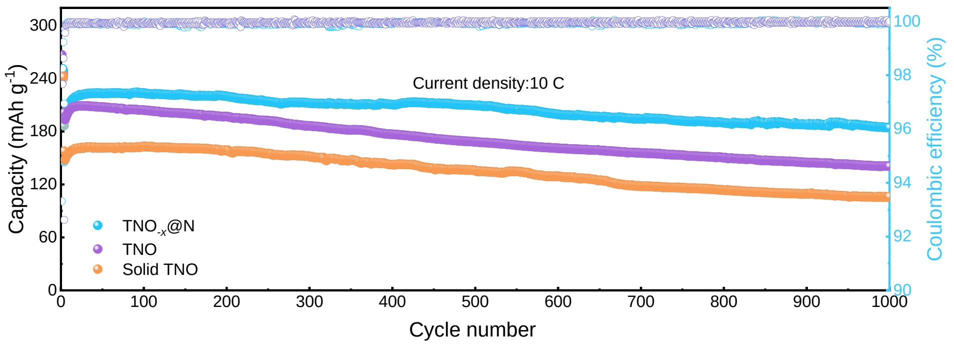

Supplementary Fig. 27. Long-term cyclic performance of the TNO$_{-x}$@N, TNO and solid TNO electrode operated at 10 C and 25 ºC after an activation process of 3 cycles at 0.5 C.

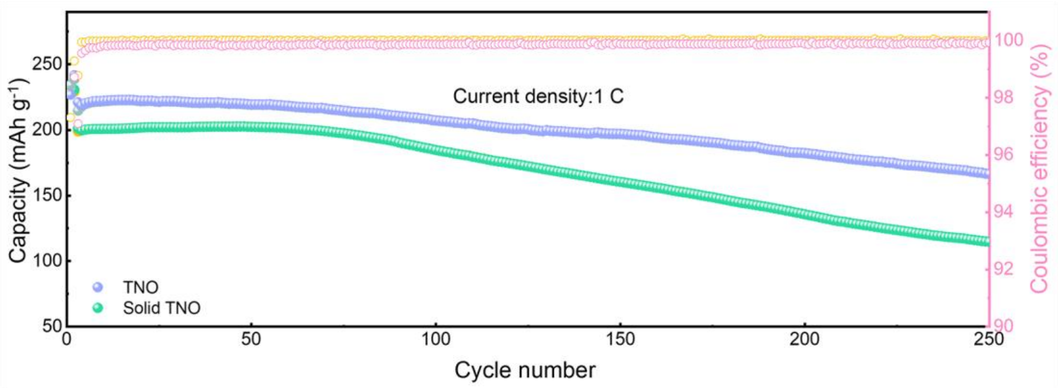

Supplementary Fig. 28. Long-term cycling stability of the TNO and solid TNO electrode with the mass loading of 10 mg cm^{-2}.

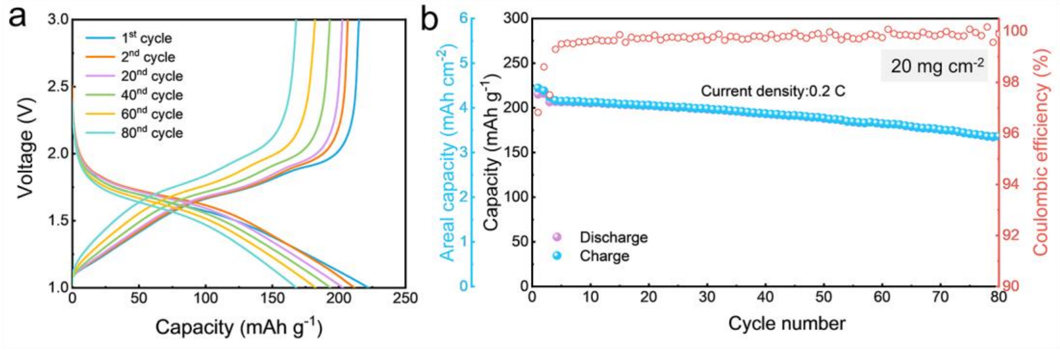

Supplementary Fig. 29. Electrochemical properties of TNO$_{-x}$@N with high active-material loadings of 20 mg cm^{-2} at 25 ℃ after an activation process of 2 cycles at 0.1 C. (a) Charge–discharge curves and (b) cyclability at 0.2 C of TNO$_{-x}$@N half coin cell.

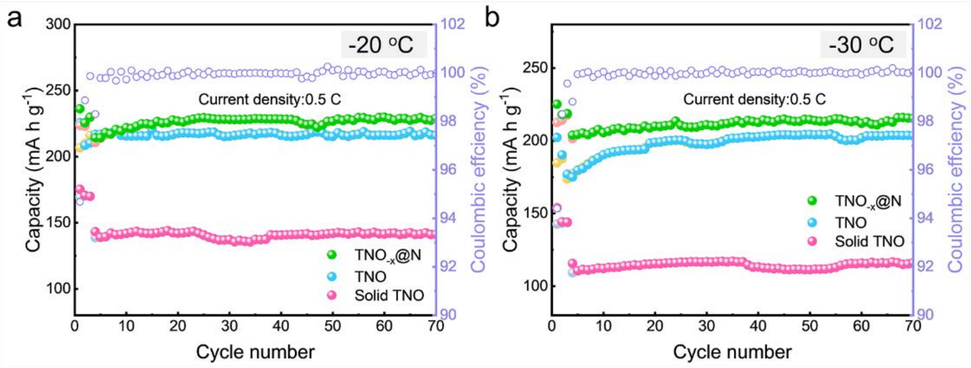

Supplementary Fig. 30. Electrochemical properties of the three electrodes at −20 °C and −30 °C. Cycling performance of the TNO_{-x}@N, TNO, and solid TNO electrode at (a) −20 °C and (b) −30 °C after an activation process of 3 cycles at 0.2 C. The average mass loading of the electrodes is about 1.5 mg cm^{-2}. (1 C=300 mA g^{-1})

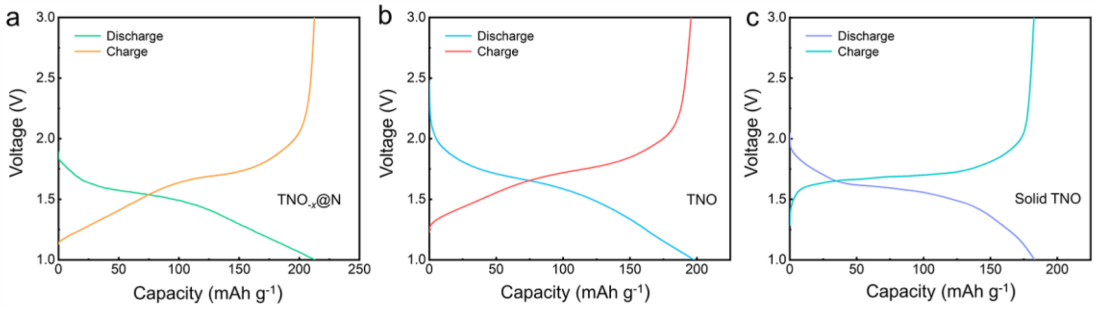

Supplementary Fig. 31. Low-temperature galvanostatic charge/discharge of (a) the TNO$_{-x}$@N, (b) TNO, and (c) solid TNO with the mass loading of 6 mg cm^{-2} at –30 °C.

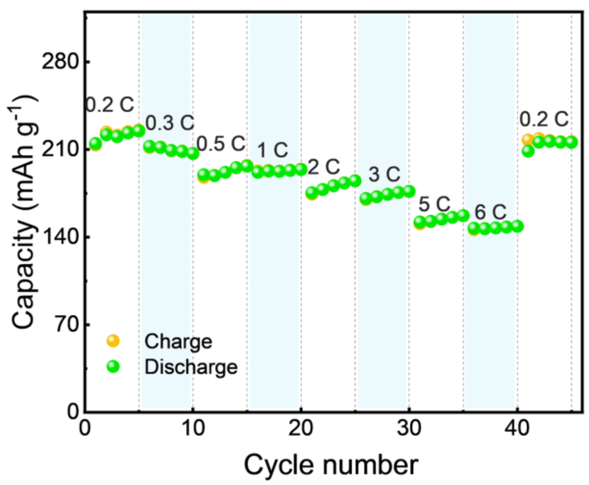

Supplementary Fig. 32. Rate performance of TNO$_{-x}$@N operated at –30 °C with the mass loading of 6 mg cm^{-2}. (1 C=250 mA g^{-1})

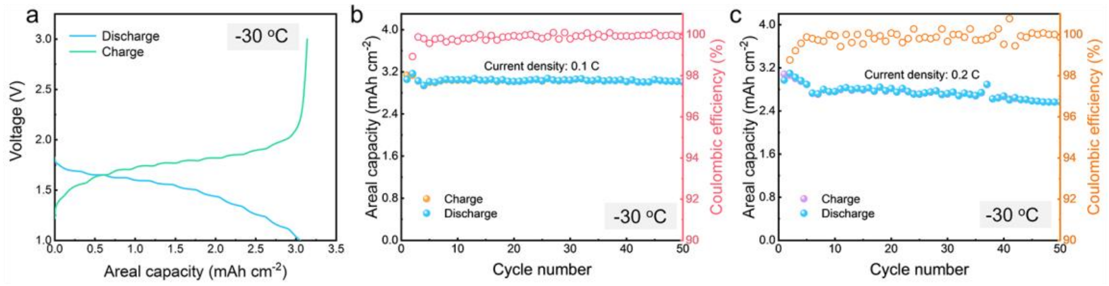

Supplementary Fig. 33. Electrochemical properties of TNO$_{-x}$@N with the high active-material loadings of 20 mg cm^{-2} at -30 °C. (a) Charge–discharge curve of TNO$_{-x}$@N, Cycling performance of TNO$_{-x}$@N half coin cell at (b) 0.1 C and (c) 0.2 C.

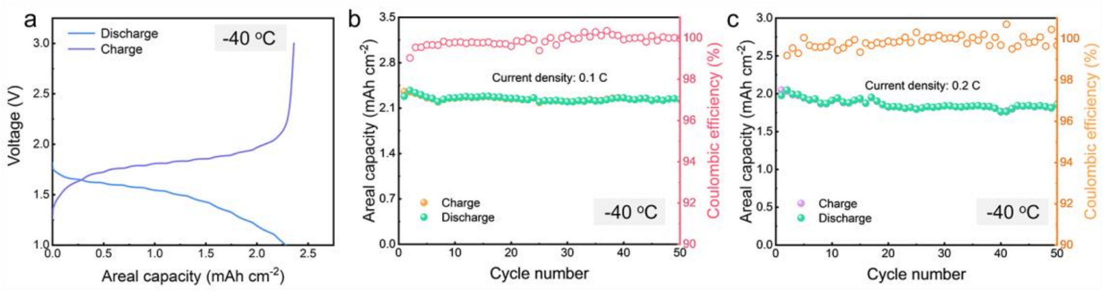

Supplementary Fig. 34. Electrochemical properties of TNO$_{-x}$@N with high active-material loadings of 20 mg cm^{-2} at -40 °C. (a) Charge–discharge curve of TNO$_{-x}$@N, Cycling performance of TNO$_{-x}$@N half coin cell at (b) 0.1 C and (c) 0.2 C.

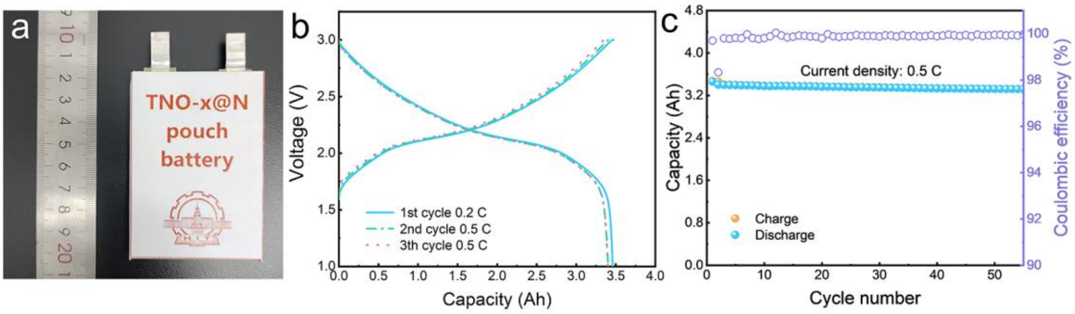

Supplementary Fig. 35. Electrochemical properties of the TNO-$_x$@N||LiNi$_{0.8}$Co$_{0.1}$Mn$_{0.1}$O$_2$ Ah-level pouch cell. (a) Schematic illustration of the TNO-$_x$@N||LiNi$_{0.8}$Co$_{0.1}$Mn$_{0.1}$O$_2$ pouch cell; (b) Charge/discharge profiles of the TNO-$_x$@N||LiNi$_{0.8}$Co$_{0.1}$Mn$_{0.1}$O$_2$ pouch cell; (c) Cycling performance the TNO-$_x$@N||LiNi$_{0.8}$Co$_{0.1}$Mn$_{0.1}$O$_2$ pouch cell at 0.5 C after an activation process of 1 cycle at 0.2 C. (1 C=3.5 A)

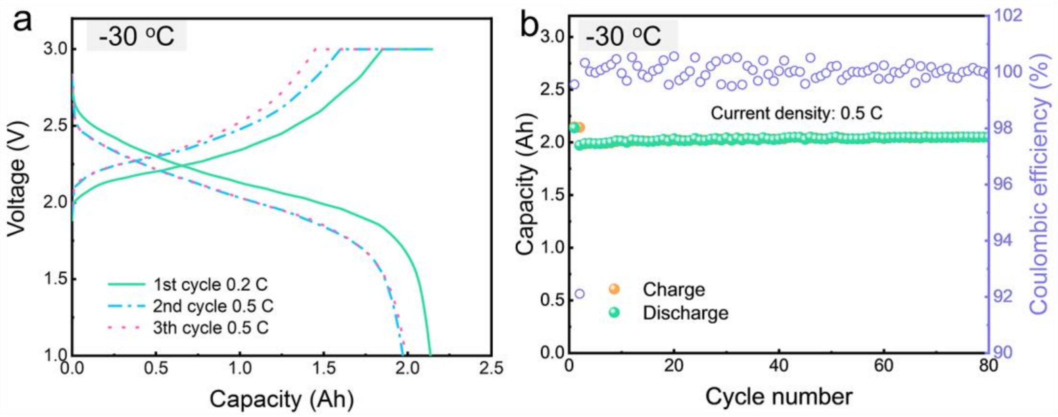

Supplementary Fig. 36. Electrochemical properties of the TNO$_{-x}$@N||$LiNi_{0.8}Co_{0.1}Mn_{0.1}O_2$ Ah-level pouch cell at −30 °C. (a) Charge/discharge profiles; (b) Cycling performance at 0.5 C after an activation process of 1 cycle at 0.2 C. (1 C=3.5 A)

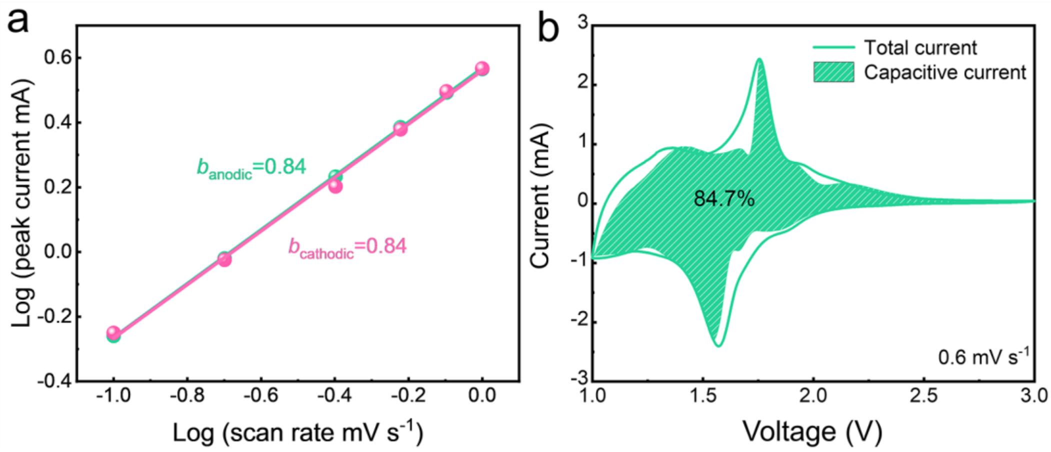

Supplementary Fig. 37. Electrochemical kinetic analysis of TNO$_{-x}$@N. (a) Log(i) versus log(v) curves (b-value determination) at 25 °C. (b) CV profiles of TNO$_{-x}$@N electrode at 0.6 mV s^{-1} and the contribution of pseudocapacitive to the total current.

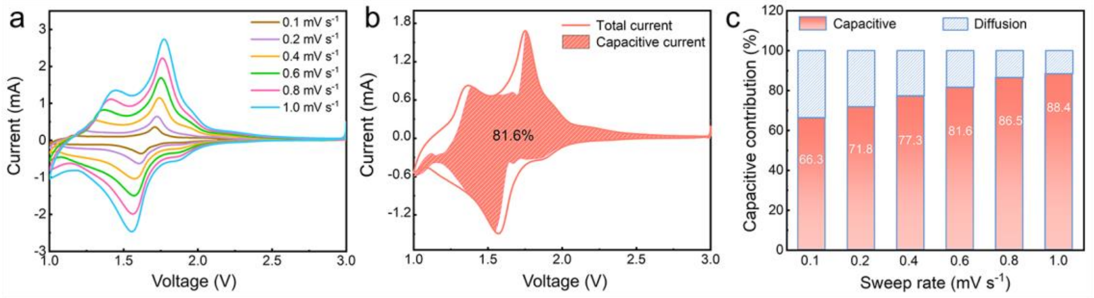

Supplementary Fig. 38. Electrochemical kinetic analysis of TNO. (a) CV curves of TNO electrode at various scan rates. (b) CV profiles of TNO electrode at 0.6 mV s^{-1} and the contribution of pseudocapacitive to the total current. (c) The percentage of pseudocapacitive contribution at different scan rates.

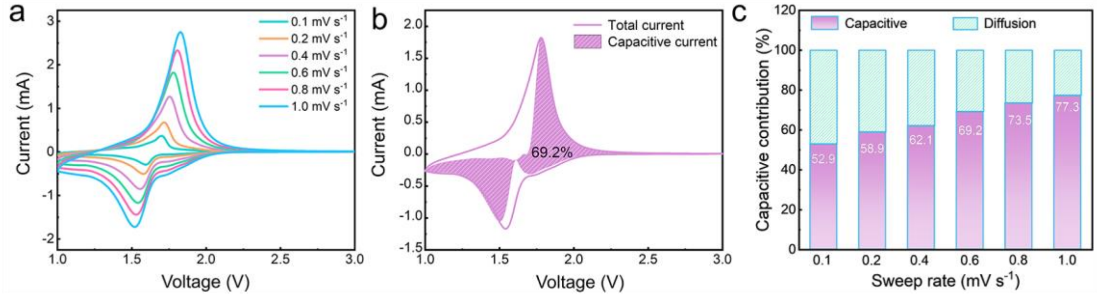

Supplementary Fig. 39. Electrochemical kinetic analysis of solid TNO. (a) CV curves of solid TNO electrode at various scan rates. (b) CV profiles of the solid TNO electrode at 0.6 mV s^{-1} and the contribution of pseudocapacitive to the total current. (c) The percentage of pseudocapacitive contribution at different scan rates.

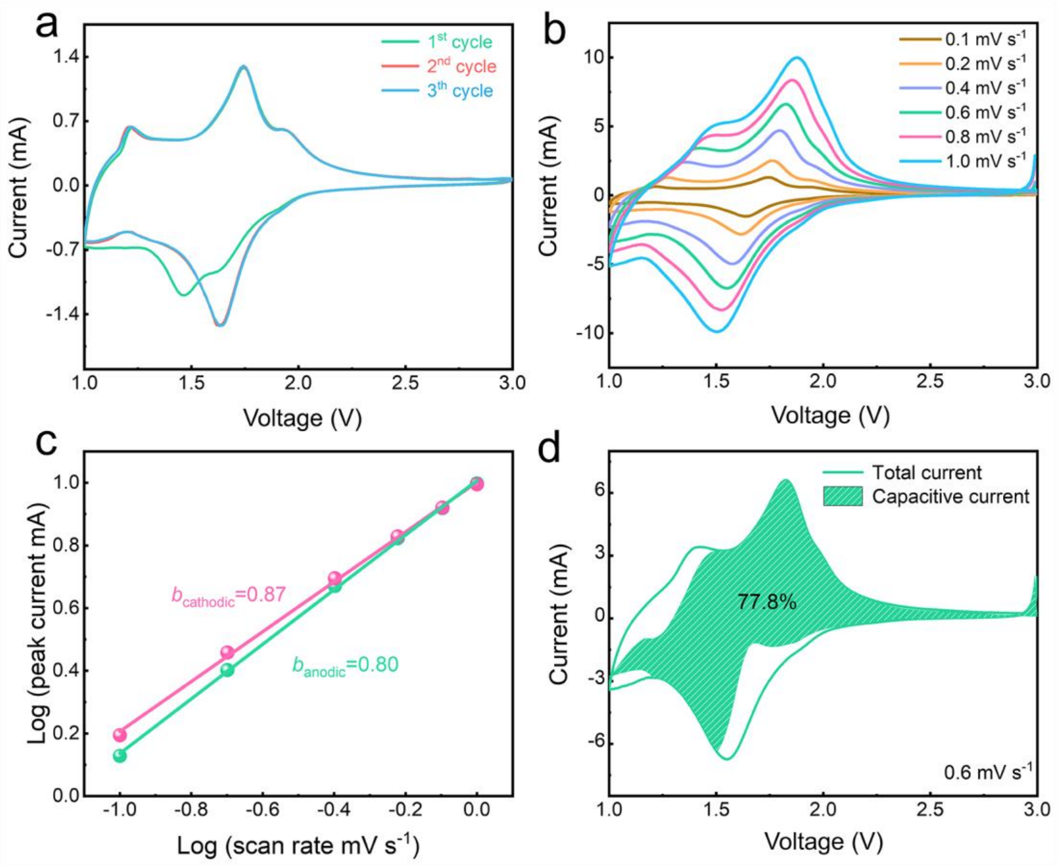

Supplementary Fig. 40. Electrochemical kinetic analysis of TNO-*x*@N with the mass loading of 8 mg cm^{-2}. (a) CV curves for the first three cycles at a scan rate of 0.1 mV s^{-1} at 25 °C. (b) CV curves at various scan rates. (c) Log(*i*) versus log(*v*) curves (*b*-value determination). (d) CV profiles of the TNO-*x*@N electrode at 0.6 mV s^{-1} and the contribution of pseudocapacitive to the total current with the high mass loading.

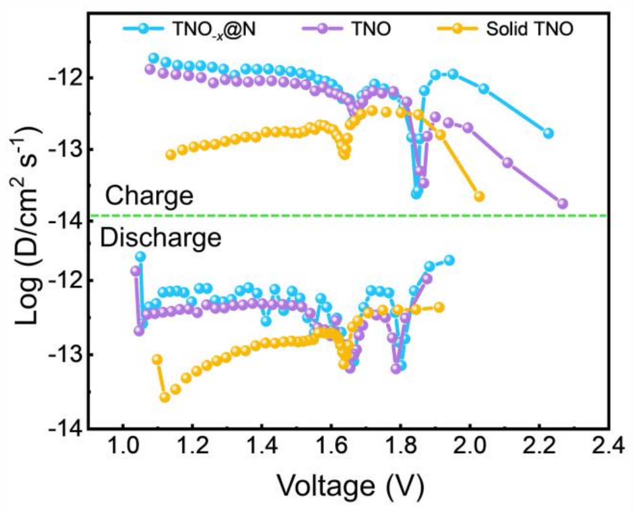

Supplementary Fig. 41. Kinetics analyses. (a) The calculated Li^{+} diffusion coefficients of the TNO_{-x}@N, TNO, and solid TNO at 25 °C.

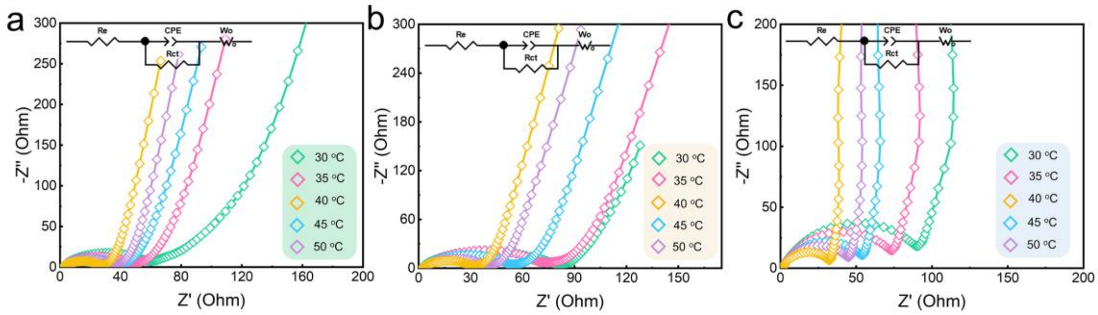

Supplementary Fig. 42. EIS spectra characterization. EIS spectra of (a) the TNO_{-x}@N, (b) TNO and (c) solid TNO electrode after the initial cycle at temperatures from 30 °C to 50 °C.

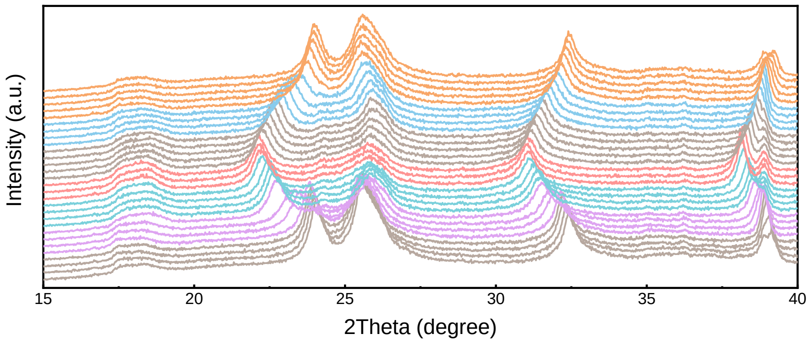

Supplementary Fig. 43. *In situ* XRD spectroscopy characterization of the TNO$_{-x}$@N electrode. Original *in situ* XRD spectra of Li||TNO$_{-x}$@N half cell during cycling.

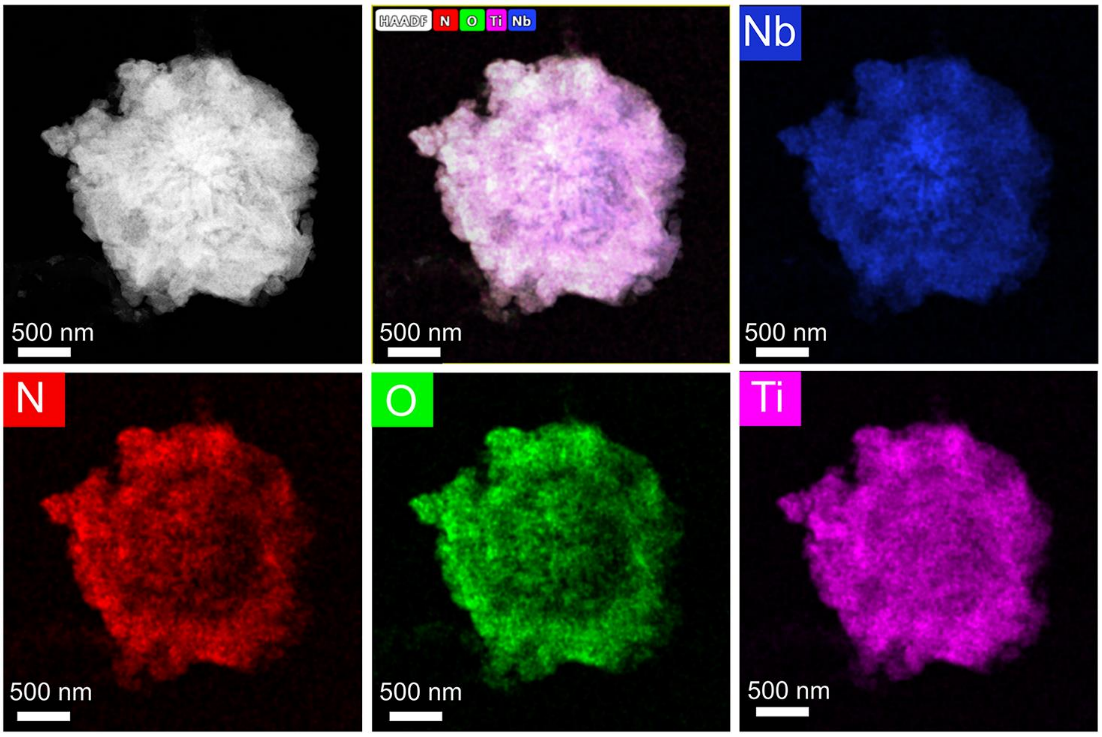

Supplementary Fig. 44. HAADF and corresponding elemental mapping images of TNO$_{-x}$@N at the charged state of 3.0 V.

Supplementary Table 1. The calculated relative energies of N doping at the different oxygen sites after structure optimization.

Site	Relative energy (eV)
**2C-1**	**0.35**
**2C-2**	**0.71**
**2C-3**	**0.64**
**3C-1**	**0.41**
**3C-2**	**0.10**
**3C-3**	**1.31**
**3C-4**	**0.52**
**4C**	**0.00**

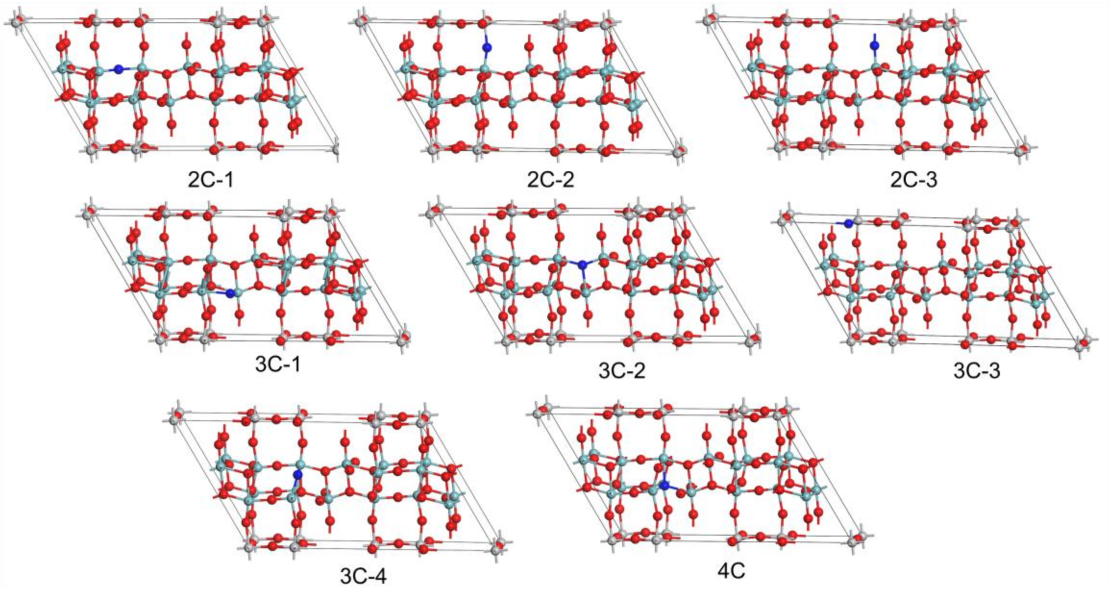

2C: Di-coordinated oxygen; 3C: Tri-coordinated oxygen; 4C: tetra-coordinated oxygen. The red, blue, cyan, and grey balls represent Nb, N, Ti, and O atoms, respectively.

Supplementary Table 2. Oxygen vacancy formation energy at different locations and with various amount.

Coordination number		1-Ovac	2-Ovac	3-Ovac	6-Ovac	9-Ovac
Di-coordinated oxygen	Ti-O-Ti	2.65	3.07	3.17		
	Ti-O-Nb	2.67	2.96	3.24		
	Nb-O-Nb	3.27	3.45	3.88		
Tri-coordinated oxygen	O-Ti3	1.43	1.53	1.81	2.21	2.79
	O-Nb3	3.9	4.21	4.61		

Supplementary Table 3. Bi-exponential dynamics fitting results of TNO and TNO_{-x}@N transient absorption dynamics at 625 nm.

Sample	τ_1 (ps)	A_1	τ_2 (ps)	A_2
TNO	1.06±0.66	37.69%	14.85±1.59	67.31%
TNO_{-x}@N	1.52±0.76	44.14%	30.22±8.72	55.86%

Supplementary Table 4. Comparisons of discharge capacity of TNO$_{-x}$@N at Low-T with previously-reported LTO- and Nb-based electrodes.

Materials	Current density (A g^{-1})/Rate (C)	Capacity (mAh g^{-1})	Loading mass (mg cm^{-2})	Reference
**TNO$_{-x}$@N**	**3 C**	**173.8 (-30 °C)**	**6**	**This work**
**TNO$_{-x}$@N**	**6 C**	**147.6 (-30 °C)**	**6**	**This work**
F-LTO	1 C	100 (-20 °C)	1.1	[1]
LTO–RTO	1 C	86 (-40 °C)	/	[2]
LTO/C	1 C	119 (-20 °C)	/	[3]
LTO-hm	5 C	107.6 (-20 °C)	2–5	[4]
LTO	0.1 C	145 (-20 °C)	~10	[5]
PR-TNO	2 C	175 (-20 °C)	/	[6]
$Ni_2Nb_{34}O_{87}$	5 C	61 (-10 °C)	1.1	[7]
W-Nb_2O_5/MXene	0.1 A g^{-1}	160.7 (-20 °C)	1	[8]

Supplementary Table 5. Comparison of the mass energy density and volumetric energy density of the TNO_{-x}@N electrode with recently reported some electrodes.

Materials	Particle size	Mass energy density (Wh kg^{-1})	Volumetric energy density (Wh L^{-1})	test types of Pouch or Coin	Refs
TNO_{-x}@N	2~3 um	271.6	373.4	pouch	**This work**
XTO	/	84	176	pouch	Toshiba
Si-C/TiO_2	5~15 µm	288.4	350.6	pouch	[9]
Graphite	/	270	/	pouch	[10]
$Nb_1Mo_{0.1}O_{2.8}$	1 um	129	146	Coin	[11]
$Li_4Ti_5O_{12}$	/	110	100	Coin	[11]
Nb_{16}/Nb_{18}	1 um	158	/	Coin	[12]
Graphite/SiN	2~3 um	/	412.2	pouch	[13]

Supplementary Table 6. Equivalent circuit parameters of the TNO_{-x}@N and TNO at 25 ºC and −40 ºC.

Material	R_{ct}/Ohm
TNO_{-x}@N (25 ºC)	90.74
TNO (25 ºC)	115.07
Solid TNO (25 ºC)	157.63
TNO_{-x}@N (−40 ºC)	445.3
TNO (−40 ºC)	588.69
Solid TNO (−40 ºC)	839.72

Supplementary Table 7. Comparisons of apparent Li^+ diffusion coefficient (D_{Li^+}) of TNO_{-x}@N at -40 °C with previously-reported M–Nb–O negative materials at 25 °C.

Material	D_{Li^+} ($cm^2\ s^{-1}$)	Test technique	Reference
**TNO_{-x}@N microflowers**	**4.0×10^{-13} (25 °C)**	**GITT**	**This work**
**TNO_{-x}@N microflowers**	**1.6×10^{-14} (-40 °C)**	**GITT**	**This work**
TNO microflowers	5.5×10^{-15} (-40 °C)	GITT	This work
Solid TNO	4.4×10^{-15} (-40 °C)	GITT	This work
$Mo_{1.5}W_{1.5}Nb_{14}O_{44}$ micron-sized particles	7.7×10^{-18} (25 °C)	GITT	[14]
$N\text{-}Nb_2O_5$ microflowers	2.4×10^{-16} (25 °C)	EIS	[15]
$Cr_{0.6}Ti_{0.8}Nb_{10.6}O_{29}$ micron-sized	1.4×10^{-14} (25 °C)	EIS	[16]
VNb_9O_{25} nanoribbons	5.2×10^{-15} (25 °C)	EIS	[17]
L-NbO sphere-like	7.3×10^{-15} (25 °C)	GITT	[18]
$GeNb_{18}O_{47}$ nanowires	1.6×10^{-14} (25 °C)	CV	[19]
m-TNO@C nanoparticles	5.7×10^{-17} (25 °C)	CV	[20]
$TiCr_{0.5}Nb_{10.5}O_{29}$ nanoparticles	2.0×10^{-14} (25 °C)	CV	[21]
$Cr_{0.5}Nb_{24.5}O_{62}$ nanowires	4.6×10^{-14} (25 °C)	EIS	[22]
Nb_2O_5 nanorods	3.6×10^{-17} (25 °C)	CV	[23]

Supplementary Table 8. Equivalent circuit parameters of the solid TNO, TNO, and TNO_{-x}@N electrode after the initial cycle at temperatures from 30 to 50 °C.

Temperature	R_{ct} (Solid TNO)	R_{ct} (TNO)	$\boldsymbol{R_{ct}}$ **(TNO_{-x}@N)**
30 ^{o}C	90.03	74.38	**48.19**
35 ^{o}C	73.04	67.13	**42.92**
40 ^{o}C	53.52	50.53	**35.32**
45 ^{o}C	44.27	36.58	**33.55**
50 ^{o}C	32.96	28.93	**25.56**

Supplementary References

1. Zhang, Y., Luo, Y., Chen, Y., Lu, T., Yan, L., Cui, X., Xie, J. Enhanced rate capability and low-temperature performance of $Li_4Ti_5O_{12}$ anode material by facile surface fluorination. *ACS Appl. Mater. Interfaces* **9**, 17145-17154 (2017).
2. Huang, Q., Yang, Z., Mao, J. Mechanisms of the decrease in low-temperature electrochemical performance of $Li_4Ti_5O_{12}$-based anode materials. *Sci. Rep.* **7**, 15292 (2017).
3. Yuan, T., Yu, X., Cai, R., Zhou, Y., Shao, Z. Synthesis of pristine and carbon-coated $Li_4Ti_5O_{12}$ and their low-temperature electrochemical performance. *J. Power Sources* **195**, 4997-5004 (2010).
4. Meng, Q., Hao, Q., Chen, F., Wang, L., Li, N., Sun, X. $Li_4Ti_5O_{12}$ hollow macrospheres combine high tap density and excellent low-temperature performance as anode materials for Li-ion batteries. *Mater Charact.* **203**, 113089 (2023).
5. Xu, J., Wang, X., Yuan, N., Ding, J., Qin, S., Razal, J. M., Wang, X., Ge, S., Gogotsi, Y. Extending the low temperature operational limit of Li-ion battery to −80 °C. *Energy Storage Mater.* **23**, 383-389 (2019).
6. Jiang, T., Ma, S., Deng, J., Yuan, T., Lin, C., Liu, M. Partially reduced titanium niobium oxide: a high-performance lithium-storage material in a broad temperature range. *Adv. Sci.* **9**, 2105119 (2022).
7. Lv, C., Lin, C., Zhao, X. S. Rational design and synthesis of nickel niobium oxide with high-rate capability and cycling stability in a wide temperature range. *Adv. Energy Mater.* **12**, 2102550 (2022).
8. Chen, Y., Pu, Z. Y., Liu, Y. B., Shen, Y. X., Liu, S. M., Liu, D., Li, Y. M. Enhancing the low-temperature performance in lithium ion batteries of Nb_2O_5 by combination of W doping and MXene addition. *J. Power Sources* **515**, 230601 (2021).
9. Xu, C., Shen, L., Zhang, W., Huang, Y., Sun, Z., Zhao, G., Lin, Y., Zhang, Q., Huang, Z., Li, J. Efficient implementation of kilogram-scale, high-capacity and long-life Si-C/TiO_2 anodes. *Energy Storage Mater.* **56**, 319-330 (2023).
10. Zheng, X., Cao, Z., Luo, W., Weng, S., Zhang, X., Wang, D., Zhu, Z., Du, H., Wang, X., Qie, L., Zheng, H., Huang, Y. Solvation and interfacial engineering enable −40 °C operation of graphite/NCM batteries at energy density over 270 Wh kg^{-1}. *Adv. Mater.* **35**, 2210115 (2023).
11. Shen, F., Sun, Z., Zhao, L., Xia, Y., Shao, Y., Cai, J., Li, S., Lu, C., Tong, X., Zhao, Y., Sun, J., Shao, Y. Triggering the phase transition and capacity enhancement of Nb_2O_5 for fast-charging lithium-ion storage. *J. Mater. Chem. A* **9**, 14534-14544 (2021).
12. Ma, J., Zhang, H., Yu, X., Xiang, Y., Qiu, J., Liu, S., Lin, H., Cao, G., Zhang, W. Regulating the local coordination model of homologous and heterogeneous niobium tungsten oxides toward ultrafast lithium storage. *Energy Storage Mater.* **63**, 102979 (2023).
13. Chae, S., Park, S., Ahn, K., Nam, G., Lee, T., Sung, J., Kim, N., Cho, J. Gas phase synthesis of amorphous silicon nitride nanoparticles for high-energy LIBs. *Energy Environ. Sci.* **13**, 1212-1221 (2020).
14. Tao, R., Zhang, T., Tan, S., Jafta, C. J., Liang, J., Sun, X.-G., Wang, T., Fan, J., Lu, Z., Bridges, C. A., Dai, S. Insight into the fast-rechargeability of a novel $Mo_{1.5}W_{1.5}Nb_{14}O_{44}$ anode material for high-performance lithium-ion batteries. *Adv. Energy Mater.* **12**, 2200519 (2022).

15. Liu, G., Liu, S., Chen, H., Liu, X., Luo, X., Li, X., Ma, J. Highly [001]-oriented N-doped orthorhombic Nb_2O_5 microflowers with intercalation pseudocapacitance for lithium-ion storage. *Nanoscale* **14**, 11710-11718 (2022).
16. Yang, C., Yu, S., Ma, Y., Lin, C., Xu, Z., Zhao, H., Wu, S., Zheng, P., Zhu, Z.-Z., Li, J., Wang, N. Cr^{3+} and Nb^{5+} co-doped $Ti_2Nb_{10}O_{29}$ materials for high-performance lithium-ion storage. *J. Power Sources* **360**, 470-479 (2017).
17. Qian, S., Yu, H., Yan, L., Zhu, H., Cheng, X., Xie, Y., Long, N., Shui, M., Shu, J. High-rate long-life pored nanoribbon VNb_9O_{25} built by interconnected ultrafine nanoparticles as anode for lithium-ion batteries. *ACS Appl. Mater. Interfaces* **9**, 30608-30616 (2017).
18. Zheng, Y., Yao, Z., Shadike, Z., Lei, M., Liu, J., Li, C. Defect-concentration-mediated T-Nb_2O_5 anodes for durable and fast-charging Li-ion batteries. *Adv. Funct. Mater.* **32**, 2107060 (2022).
19. Ran, F., Cheng, X., Yu, H., Zheng, R., Liu, T., Li, X., Ren, N., Shui, M., Shu, J. Nano-structured $GeNb_{18}O_{47}$ as novel anode host with superior lithium storage performance. *Electrochim. Acta* **282**, 634-641 (2018).
20. Qian, R., Yang, C., Ma, D., Li, K., Feng, T., Feng, J., Pan, J. H. Robust lithium storage of block copolymer-templated mesoporous $TiNb_2O_7$ and $TiNb_2O_7$@C anodes evaluated in half-cell and full-battery configurations. *Electrochim. Acta* **379**, 138179 (2021).
21. Hu, L., Lu, R., Tang, L., Xia, R., Lin, C., Luo, Z., Chen, Y., Li, J. $TiCr_{0.5}Nb_{10.5}O_{29}$/CNTs nanocomposite as an advanced anode material for high-performance Li^+-ion storage. *J. Alloys Compd.* **732**, 116-123 (2018).
22. Yang, C., Yu, S., Lin, C., Lv, F., Wu, S., Yang, Y., Wang, W., Zhu, Z.-Z., Li, J., Wang, N., Guo, S. $Cr_{0.5}Nb_{24.5}O_{62}$ nanowires with high electronic conductivity for high-rate and long-life lithium-ion storage. *ACS Nano* **11**, 4217-4224 (2017).
23. Shi, C., Xiang, K., Zhu, Y., Zhou, W., Chen, X., Chen, H. Box-implanted Nb_2O_5 nanorods as superior anode materials in lithium ion batteries. *Ceram. Inter.* **43**, 12388-12395 (2017).
